# Intravascular large B-cell lymphoma presenting with fever and refractory acidosis

**DOI:** 10.4322/acr.2021.324

**Published:** 2021-09-03

**Authors:** Michael Harrison Storandt, Mark Alan Koponen

**Affiliations:** 1 University of North Dakota School of Medicine and Health Sciences (UNDSMHS), Department of Pathology, Grand Forks, ND, USA

**Keywords:** Lymphoma, Non-Hodgkin, lactic acidosis

## Abstract

Intravascular large B-cell lymphoma (IVLBCL) is a rare form of diffuse large B-cell lymphoma, characterized by malignant B-cells primarily localized to the lumina of small- and medium-sized blood vessels without lymphadenopathy. Two patients initially presented with fever of unknown origin and persistent lactic acidosis without evidence of tissue hypoxia. Neither patient had an identifiable source of infection and both underwent peripheral blood smear demonstrating leukocytosis with a neutrophilic predominance and thrombocytopenia without evidence of hematologic malignancy. One had previously had a bone marrow biopsy which was unremarkable. Both patients’ condition deteriorated rapidly, progressing to multiorgan failure requiring pressors and mechanical ventilation, which ultimately resulted in cardiopulmonary arrest. At autopsy, each patient demonstrated malignant lymphocytoid cells, staining positive for CD20, localized to the lumina of small- and medium-sized vessels in multiple organs, including the lungs, liver, spleen, and kidneys, among others, allowing for the diagnosis of IVLBCL. IVLBCL is exceedingly rare, which in combination with significant variability in presentation, can make identification and diagnosis challenging. Diagnosis requires biopsy, therefore a high index of suspicion is needed to obtain an adequate tissue sample, whether pre- or postmortem. In the presented cases, both patients exhibited type B lactic acidosis with an unknown etiology that was ultimately determined at autopsy.

## INTRODUCTION

Intravascular large B-cell lymphoma (IVLBCL), first characterized by Pfleger and Tappeiner^1^ in 1959, is a rare form of diffuse large B-cell lymphoma, characterized by malignant B-cells primarily localized to the lumina of small- and medium-sized blood vessels without lymphadenopathy. IVLBCL is exceedingly rare, with one study demonstrating an incidence of 0.095 cases per 1,000,000.^2^ Diagnosis can be challenging due to non-specific presentation, and the disease can be rapidly fatal. We present a series of two patients presenting with fevers and persistent lactic acidosis who were ultimately diagnosed with IVLBCL at autopsy.

## CASE REPORT

### Case 1

A 66-year-old male with an unremarkable past medical history was initially admitted following cystoscopy due to benign prostatic hyperplasia resulting in urinary retention and worsening renal function. At this point, he endorsed a three-month history of malaise, fatigue, intermittent fevers, and rash, and had previously been found to be mildly anemic with splenomegaly. In the weeks prior to his current admission, he had undergone a bone marrow biopsy which did not demonstrate evidence of hematological malignancy. He was started on meropenem and linezolid due to fever with an unknown source, with suspicion of bacterial prostatitis, in spite of negative cultures. He was later treated with a 3-day course of methylprednisolone due to suspicion of Still’s disease, but this did not lead to improvement of symptoms. Over the next couple days, he was found to be retaining fluid and his creatinine continued to rise. On hospital day 6, he underwent renal biopsy which demonstrated acute tubular necrosis. The following day he developed an episode of hypotension which was responsive to intravenous fluid resuscitation, however, his lactic acid increased from 1.8 to 3.1 mEq/L (reference range [RR]; 0.5-2.2 mEq/L). Additionally, his LDH was 491 U/L (RR; 125-245 U/L). In the following days his renal function continued to deteriorate, and his lactic acid continued to rise in spite of no clinical evidence of shock. Due to worsening renal function and anion gap metabolic acidosis, he was initiated on continuous renal replacement therapy (CRRT). On hospital day 10, his lactic acid reached 12.6 mEq/L. Abdominal and pelvic computed tomography (CT) with angiography was negative for any sign of bowel ischemia. AST and ALT were within normal limits and alkaline phosphatase was elevated at 277 IU/L (RR; 30-150 IU/L). Bilirubin was mildly elevated at 1.7 mg/dL (RR; 0.2-1.2 mg/dL). At this point he had developed hypotension requiring pressors. A peripheral smear was obtained to evaluate for a hematological explanation of his elevated lactic acid as work-up for an anoxic source had been unable to elicit an etiology. Peripheral smear demonstrated mild leukocytosis with absolute neutrophilia and borderline absolute monocytosis with moderate thrombocytopenia. There was no significant dysplasia seen. On hospital day 11, the patient’s condition rapidly deteriorated. His lactic acid had risen to 27 mEq/L and he developed acute hypoxic respiratory failure requiring mechanical ventilation. He was coagulopathic with an INR of 2 (RR; <1.1). On hospital day 12, the patient’s white count was elevated at 31.0 K/uL (RR; 4.0-11.0 K/uL) with a neutrophilic predominance, hemoglobin was 10.2 g/dL (RR; 13.5-17.5 g/dL), platelet count was 19,000/uL (RR; 140-400 K/uL), and INR was 8.2. Due to his refractory shock with multiorgan failure, his family decided to make the patient a modified do not resuscitate. He later developed pulseless electrical activity which progressed to asystole. The family consented to autopsy in lieu of his refractory lactic acidosis and unknown cause of death.

### Case 2

An 82-year-old female with past medical history of hyperparathyroidism, hypertension, hyperlipidemia, chronic kidney disease stage 2, and depression presented with a one-week history of weakness, abdominal pain, and fever and was subsequently admitted. She had been treated for a urinary tract infection prior to admission. At the time of admission, she exhibited hyponatremia, low free T4 with normal TSH, and urinalysis without indication of urinary tract infection. Further work-up demonstrated borderline low cortisol, with a normal cosyntropin stimulation test. Her hospital course was further complicated by hypotension requiring vasopressors on two occasions, persistent lactic acidosis, acute on chronic kidney disease with worsening anasarca, and worsening thrombocytopenia with coagulopathy. She underwent an extensive work-up to determine a possible source of infection including CT abdomen/pelvis, which demonstrated a 5.1 cm left adnexal lesion, large gallstones, and diverticulosis. The hepatitis panel was negative; and a transthoracic echocardiogram failed to demonstrate valve vegetations. She underwent further work-up with a pelvic ultrasound which demonstrated a complex cystic mass in her left ovary. Due to worsening thrombocytopenia, she underwent peripheral smear on hospital day 13 which demonstrated mild leukocytosis with a white cell count of 18.02 K/uL, mild neutrophilia with an absolute neutrophil count of 16218/uL, mild normochromic normocytic anemia with a hemoglobin of 10.5 g/dL, and moderate thrombocytopenia with a platelet count of 55,000/uL. There was no evidence of malignancy. On hospital day 14, due to anasarca, acidosis, and continued worsening of her renal function, CRRT was initiated. The following day, CT chest/abdomen/pelvis was obtained to again look for an infectious source of her worsening shock and worsening acidosis. This did demonstrate new patchy infiltrates in the upper lung lobes which were thought to indicate infection, and she was started on broad spectrum antibiotics. Over the course of this day, she became progressively more lethargic and developed acute hypoxic respiratory failure requiring endotracheal intubation. At this point, her lactic acid had increased from 4 to 8 mEq/L and she was requiring increased doses of pressors. By hospital day 15, she was in multiorgan failure with lactic acidosis and hypotension requiring pressors. The etiology was unknown without evidence for underlying infection. There was concern of ovarian cancer considering the complex cystic mass involving her left adnexa. Due to the patient’s poor condition and low likelihood of recovery, based on previous stated wishes of the patient, the family elected to discontinue mechanical ventilation and the patient passed away within half-an-hour. The family did consent to autopsy to determine a cause of death.

## AUTOPSY PRESENTATIONS

### Case 1

**Figure 1:**
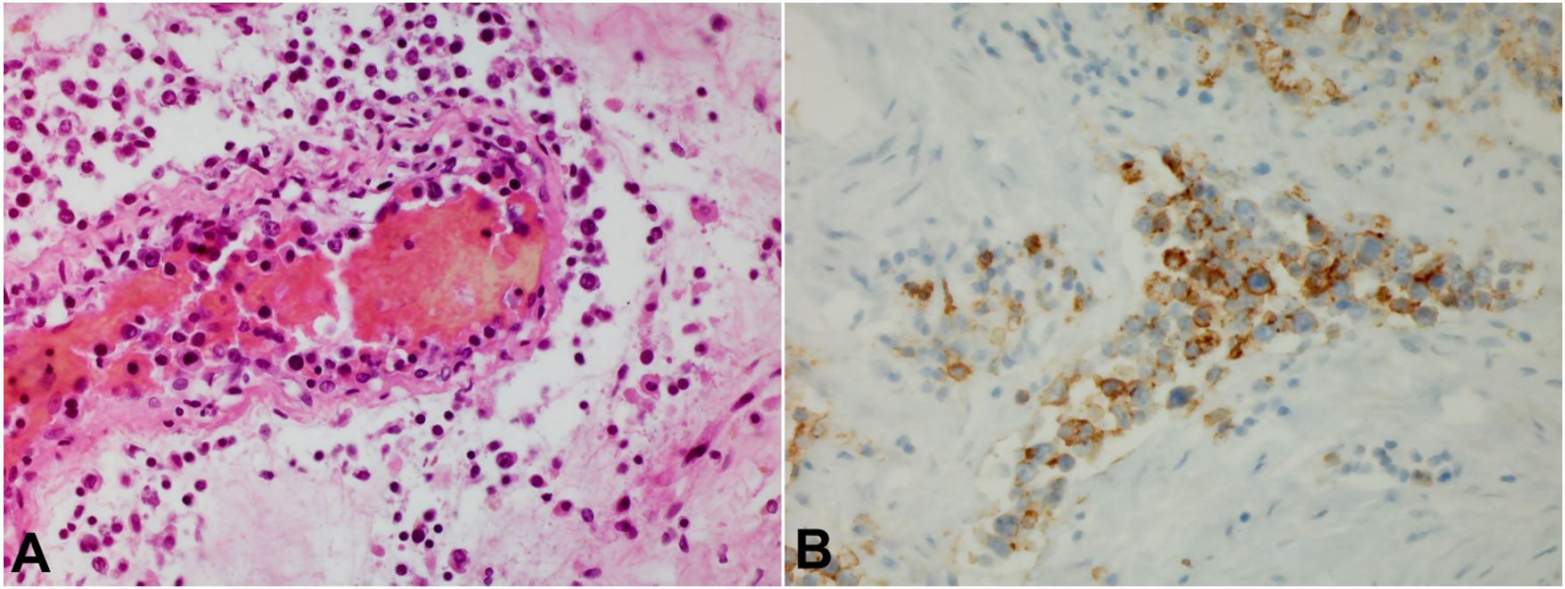
**A** – Medium sized blood vessel, Kidney (H&E, 440x); **B** – CD20 immunostaining, Kidney (440x) demonstrating malignant lymphoid infiltrate which stains positive for CD20.

Internal and external gross examination was unrevealing as to a cause of death. Histological examination did reveal acute tubular necrosis most likely related to an episode of hypotension and a resolving pneumonia; however, these would not explain the patient’s lactic acidosis and shock state. Examination of multiple organs did show malignant lymphoid cells with enlarged round or oval nuclei with prominent chromatin and occasional nucleoli and scant cytoplasm within the vascular spaces (Figure 1A). These malignant cells did stain positive for CD20 (Figure 1B), and were found in vasculature of the brain, heart, lungs, liver, spleen, kidneys, thyroid, peri-adrenal adipose, prostate, and testes. Bone marrow and abdominal lymph nodes did not contain any malignant cells. Dissemination of malignant B-cells within the vasculature of multiple organs was sufficient for diagnosis of intravascular large B-cell lymphoma.

### Case 2

**Figure 2:**
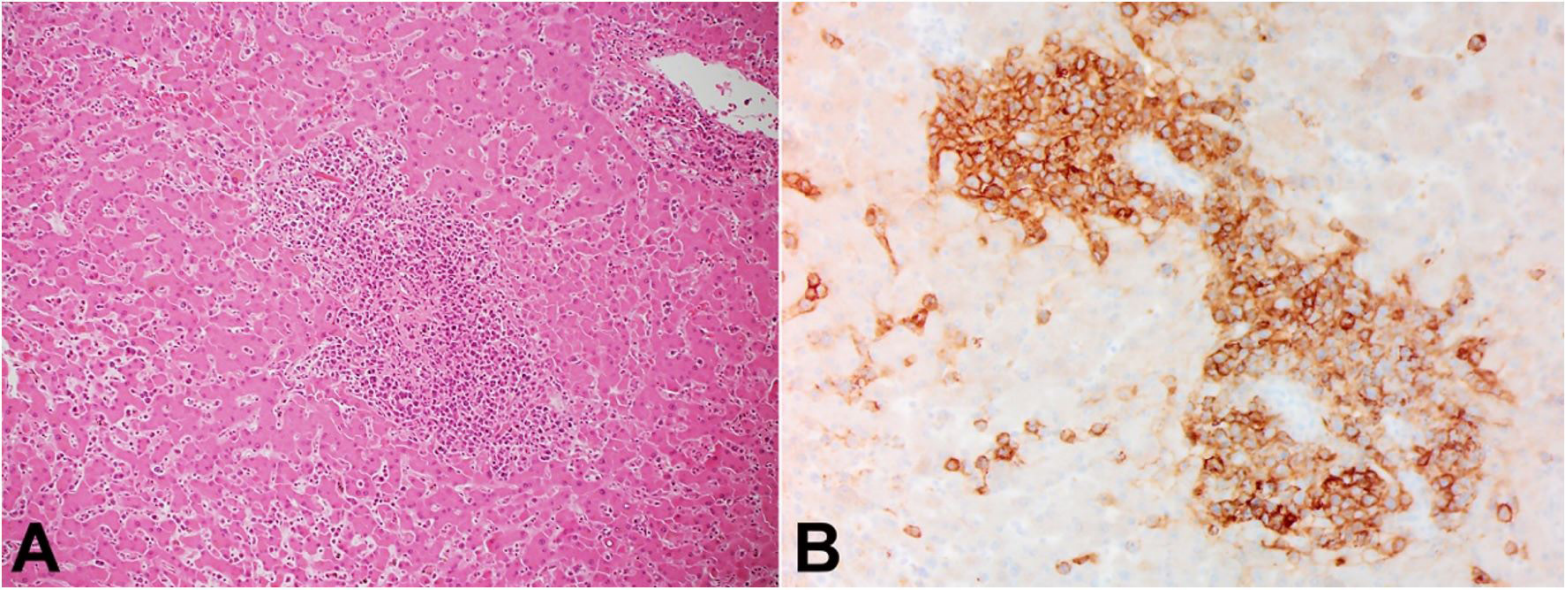
**A** – Liver with lymphocytic infiltrate (H&E, 440x); **B** – CD20 immunostaining, Liver (440x) demonstrating malignant lymphoid infiltrate which stains positive for CD20.

Initial gross external and internal examination of the body did not reveal an apparent cause of death. Histological examination was conducted which found the left ovarian mass, previously concerning for malignancy, to be a simple cyst. Sections of the lungs, liver, spleen, kidneys, and periadrenal adipose demonstrated intravascular infiltrates of malignant lymphocytoid cells with enlarged nuclei with irregular chromatin and often prominent nucleoli within the vasculature. These cells stained positive for CD20 (Figure 2) and negative for CD3 and CD34. Cervical lymph nodes sampled did not contain malignant cells. The bone marrow demonstrated essentially 100% cellularity composed largely of lymphocytoid cells, which showed the same immunohistochemical staining pattern. This was sufficient for diagnosis of intravascular large B-cell lymphoma.

## DISCUSSION

IVLBCL can manifest in many different ways making diagnosis challenging. The median age at diagnosis is 67-70 years.^2^^,^^3^ There are two primary clinical variants which are largely dependent on geography, although exceptions to these trends are not uncommon.^4^ In Western countries, patients more commonly present with cutaneous and neurological symptoms, whereas patients in Asian countries more frequently exhibit a hemophagocytic syndrome, manifesting as fever, hepatosplenomegaly, and thrombocytopenia; although generally speaking, disease manifestation is primarily related to organ involvement.^4^^,^^5^ Renal involvement has been infrequently reported, however, both cases detailed above exhibited both clinical and histological evidence of renal involvement.^6^ Additionally, patients can rapidly progress to exhibit multi-organ failure, such as seen in the presented case.^7^

However, as these symptoms are nonspecific, and this condition is exceedingly rare, clinical diagnosis can be difficult. In both cases, the patient developed persistent lactic acidosis in the absence of obvious tissue hypoxia. There are two types of lactic acidosis: type A and type B. Type A lactic acidosis stems from hypoxemia and is far more common, whereas type B acidosis stems from non-hypoxic causes, which may include toxins, side-effects of various drugs (such as metformin), liver disease, total parenteral nutrition, HIV, thiamine deficiency, trauma or excessive exercise, diabetic ketoacidosis, mitochondrial myopathy, ethanol use, and malignancy.^8^^,^^9^ Type B lactic acidosis stemming from malignancy is most commonly associated with hematologic malignancies.^10^ Multiple theories exist as to why this occurs, including increased pyruvate production, thiamine depletion, or increased rates of glycolysis in tumor cells.^10^ As demonstrated in the presented case, identification of type B lactic acidosis is often delayed as type A is far more common and is often ruled out first.

Diagnosis of IVLBCL, whether in a clinical setting or at autopsy, requires biopsy of an affected organ, which is commonly the skin in living patients with cutaneous manifestations. Histological findings seen in IVLBCL include lymphocytoid cells located within small- and medium-sized vessels with round nuclei, vesicular chromatin, and prominent nucleoli.^5^ These cells will stain positively for B-cell markers, including CD20.^11^^,^^12^ It is not yet known why these cells preferentially localize to small- and medium-sized blood vessels, although it is thought to be related to aberrant expression of cell surface proteins that result in sequestration of malignant B-cells within blood vessels, while also lacking expression of proteins allowing for migration through the vessel walls.^13^ Patients who are diagnosed with IVLBCL are commonly treated with rituximab and cyclophosphamide, doxorubicin, vincristine, and prednisone (CHOP) therapy, and 5 year survival for patients undergoing treatment may be as high as 60%.^14^

## CONCLUSIONS

IVLBCL is a very rare condition with diagnosis requiring biopsy, whether pre- or postmortem. It can progress rapidly and lead to multiorgan failure, as demonstrated in the presented case. It is essential that providers be aware of this rare condition, and that pathologists understand its histological presentation to allow for diagnosis. At autopsy, diagnosis may only be accomplished by histological sectioning of the affected organs.

## References

[1] Pfleger L, Tappeiner J (1959). On the recognition of systematized endotheliomatosis of the cutaneous blood vessels (reticuloendotheliosis?). Hautarzt.

[2] Rajyaguru DJ, Bhaskar C, Borgert AJ, Smith A, Parsons B (2017). Intravascular large B-cell lymphoma in the United States (US): a population-based study using Surveillance, Epidemiology, and End Results program and National Cancer Database. Leuk Lymphoma.

[3] Murase T, Yamaguchi M, Suzuki R (2007). Intravascular large B-cell lymphoma (IVLBCL): a clinicopathologic study of 96 cases with special reference to the immunophenotypic heterogeneity of CD5. Blood.

[4] Ponzoni M, Campo E, Nakamura S (2018). Intravascular large B-cell lymphoma: a chameleon with multiple faces and many masks. Blood.

[5] Ponzoni M, Ferreri AJM, Campo E (2007). Definition, diagnosis, and management of intravascular large B-cell lymphoma: proposals and perspectives from an international consensus meeting. J Clin Oncol.

[6] Desclaux A, Lazaro E, Pinaquy J, Yacoub M, Viallard J (2017). Renal intravascular large B-cell lymphoma: a case report and review of the literature. Intern Med.

[7] Ronny FMH, Black MA, Arbini AA (2017). Intravascular large B-cell lymphoma with multi-organ failure presenting as a pancreatic mass: a case with atypical presentation and definite diagnosis postmortem. Autops Case Rep.

[8] Dean RK, Subedi R, Gill D, Nat A (2017). Consideration of alternative causes of lactic acidosis: thiamine deficiency in malignancy. Am J Emerg Med.

[9] Foucher CD, Tubben RE, Abai B (2021). StatPearls..

[10] Liu QS, Harji F, Jones A, Tarnower AC (2020). Type B lactic acidosis: a rare oncological emergency. BMJ Case Rep.

[11] Estalilla OC, Koo CH, Brynes RK, Medeiros LJ (1999). Intravascular large B-cell lymphoma. A report of five cases initially diagnosed by bone marrow biopsy. Am J Clin Pathol.

[12] Khalidi HS, Brynes RK, Browne P, Koo CH, Battifora H, Medeiros LJ (1998). . Intravascular large B-cell lymphoma: the CD5 antigen is expressed by a subset of cases. Mod Pathol.

[13] Orwat DE, Batalis NI (2012). Intravascular large B-cell lymphoma. Arch Pathol Lab Med.

[14] Matsue K, Abe Y, Narita K (2019). Diagnosis of intravascular large B cell lymphoma: novel insights into clinicopathological features from 42 patients at a single institution over 20 years. Br J Haematol.

